# Screening of Small Molecule Microarrays for Ligands Targeted to the Extracellular Epitopes of Living Cells

**DOI:** 10.3390/microarrays4010053

**Published:** 2015-02-12

**Authors:** Jeong Heon Lee, Kai Bao, John V. Frangioni, Hak Soo Choi

**Affiliations:** 1 Division of Hematology/Oncology, Department of Medicine, Beth Israel Deaconess Medical Center and Harvard Medical School, Boston, MA 02215, USA; E-Mails: jlee27@bidmc.harvard.edu (J.H.L.); kbao@bidmc.harvard.edu (K.B.); jfrangio@bidmc.harvard.edu (J.V.F.); 2 Curadel, LLC, 377 Plantation Street, Worcester, MA 01605, USA; 3 Department of Cogno-Mechatronics Engineering, Pusan National University, Busan 609-735, Korea

**Keywords:** small molecules, high-throughput screening, live cells, microarrays, cell‑based assay, drug discovery

## Abstract

The screening of living cells using high-throughput microarrays is technically challenging. Great care must be taken in the chemical presentation of potential ligands and the number of collisions that cells make with them. To overcome these issues, we have developed a glass slide-based microarray system to discover small molecule ligands that preferentially bind to one cell type over another, including when the cells differ by only a single receptor. Chemical spots of 300 ± 10 μm in diameter are conjugated covalently to glass slides using an arraying robot, and novel near-infrared fluorophores with peak emission at 700 nm and 800 nm are used to label two different cell types. By carefully optimizing incubation conditions, including cell density, motion, kinetics, detection, *etc.* we demonstrate that cell-ligand binding occurs, and that the number of cells bound per chemical spot correlates with ligand affinity and specificity. This screening system lays the foundation for high-throughput discovery of novel ligands to the cell surface.

## 1. Introduction

Extracellular membrane (ECM) receptors are of significant importance to the development of new therapeutic agents, being the molecular targets for more than 60% of clinical drugs [1,2]. To discover new potential targets and to identify therapeutic agents, various diversity-oriented combinatorial libraries have been developed [3,4,5,6]. The small molecule microarray (SMM) is one of the most effective profiling solutions through the use of pre-patterned regions of interest (ROI), which provide chemical identity and functionality [7,8,9,10,11]. Although having significant potential, high-throughput SMM screening is currently limited due to the use of non-physiological contexts (e.g., absence of serum) and non-viable samples (e.g., cell lysates); most current methods also neglect ECM dynamics [12,13,14]. Microfluidics is a new technology that enables many assays using living cells, but it is not conducive to high-throughput chemical screening [15,16,17,18].

A simple alternative approach is direct panning of living cells over ligand-spotted microarrays, where each spot is a single, defined chemical entity. We have previously described functionalized microarray slides capable of rapid and high-throughput screening of over 5000 different chemical compounds binding to living bacteria, including quantitation of binding parameters [19,20,21]. In this study, we optimized the key experimental parameters for screening living mammalian cells using known small molecule ligands on the previously developed SMM, which requires simultaneous optimization of ligand presentation, the effect of motion, incubation time, ligand concentration, and the number of panned cells.

## 2. Experimental Section

### 2.1. Chemicals and Microarray

All chemicals were American Chemical Society grade or higher and commercially available unless noted otherwise. We previously provided details on the microarray slide, which uses *N*‑hydroxysuccinimide modified polyethylene glycol (PEG-NHS) for both conjugation of potential ligands and presentation of ligands far from the glass surface [20]. All ligands were engineered to contain a single nucleophilic group (*i.e.*, primary amine) and were dissolved as stock solutions in 70%/30% (V/V) glycerol:dimethyl sulfoxide (DMSO, Sigma, St Louis, MO, USA) at a concentration of 0.1–1.0 mM. The pH was adjusted to be ≈ 9.0 using diisopropylethylamine (DIEA, Sigma) to promote nucleophilic substitution using the primary amine. Polyallrylamine (PAAm, Sigma) was used as a positive control for cell binding at 10 mg/mL in 100 mM sodium bicarbonate buffer (pH 9.0). Cyclo Arg-Gly-Asp-D-Tyr-Lys (cRGDyk) peptide was purchased from AnaSpec, Inc. (Fremont, CA, USA). 2[(3-amino-3-carboxypropyl)(hydroxy)(phosphinyl)-methyl]pentane-1,5-dioic acid (GPI), 2-(3-(5-amino-1-carboxypentyl)ureido)pentanedioic acid (KUE), beta Ala-Gly (β-AG) and all trimeric ligands were in house compounds, which were synthesized as previously described [22,23]. The ligand solution was distributed into a 384-well plate and printed on the NHS functionalized slide surface [20] using a microarray robot (OmniGrid Accent, DigiLab, Inc., Holliston, MA, USA) mounted with SMP11 pins (Telechem, Sunnyvale, CA, USA). The spreading of spots was optimized to be 300 ± 10 μm in diameter, with a center-to-center spacing of 500 μm (*i.e.*, 200-μm of clear space between any two spots). After incubating for 3 h at room temperature in air, the slides were immersed in deionized water to deactivate unreacted NHS groups. After washing with ethanol, the slides were dried under a nitrogen stream and stored in a dust free environment before use.

### 2.2. Cell Culture and Adhesion Assay

Melanoma cell lines including M21, M21-L, and B16 were grown in DMEM supplemented with 10% fetal bovine serum (FBS) and 100 units/mL penicillin/streptomycin (P/S) under 5% CO_2_ at 37 °C. Human prostate cancer cells, LNCaP and PC3, were cultured in RPMI supplemented with 10% FBS and 100 units/mL P/S under 10% CO_2_ at 37 °C. All cells were plated into 100-mm culture dishes (Corning, Tewksbury, MA, USA) in 10 mL of pre-warmed culture media. When the cells reached 70%–80% confluence at a density of 2–5 × 10^6^ cells/dish, either ESNF10 (700 nm NIR fluorophore) [24] or IR786 (800 nm NIR fluorophore) [21] was added to the dish at 2 µM in media. After 20 min incubation at 37 °C, the cells were washed twice with media and the NIR fluorescence signals were observed under a multi-channel fluorescence microscope (see below). The cells were then trypsinized and seeded onto the ligand-bearing surface in DMEM containing 10% FBS at 37 °C. Incubation parameters, including the effect of rocking (0 *vs.* 30 rpm), incubation time (30–180 min), presented ligand concentration at the time of spotting (0.1–1.0 mM), and applied cell density (0.2 × 10^6^−8 × 10^6^ cells), were systematically optimized. After incubation, the slides were gently washed with cell culture media before scoring.

### 2.3. Fluorescence Microscopy and Software

Living cells bound to chemical spots were imaged using a Nikon TE2000 epifluorescence microscope equipped with a 75 W Xenon light source and an Orca-ER (Hamamatsu, Bridgewater, NJ, USA) camera [25,26]. Two custom filter sets (Chroma Technology Corporation, Brattleboro, VT, USA) composed of 650 ± 22 nm and 750 ± 25 nm excitation filters, 675 nm and 785 nm dichroic mirrors, and 710 ± 25 nm and 810 ± 20 nm emission filters were, respectively, used to detect ESNF10 (700 nm, pseudo-colored in red) and IR786 (800 nm, pseudo-colored in lime green) emission. For high-throughput imaging of microarrays, we have previously developed an automated microscope stage and software [21]. The complete scanning time for one microarray slide containing 5076 spots was approximately 2 h (1 s per spot plus stage movement time) using the automated microscope. IPLab 3.6 software (Nikon Inc., Melville, NY, USA) and ImageJ 1.45q (NIH, Bethesda, MD, USA) were used for normalization and autosegmentation of the fluorescence intensity of each spot. Sequential procedures for scoring were defined through region-of-interest (ROI) selection, static thresholding, binary image, and auto-counting. Data plotting was performed using Prism version 4.0a software (GraphPad, San Diego, CA, USA) and Microsoft Excel (Redmond, WA, USA).

## 3. Results and Discussion

### 3.1. Live Cell Imaging and Controls

**Figure 1:**
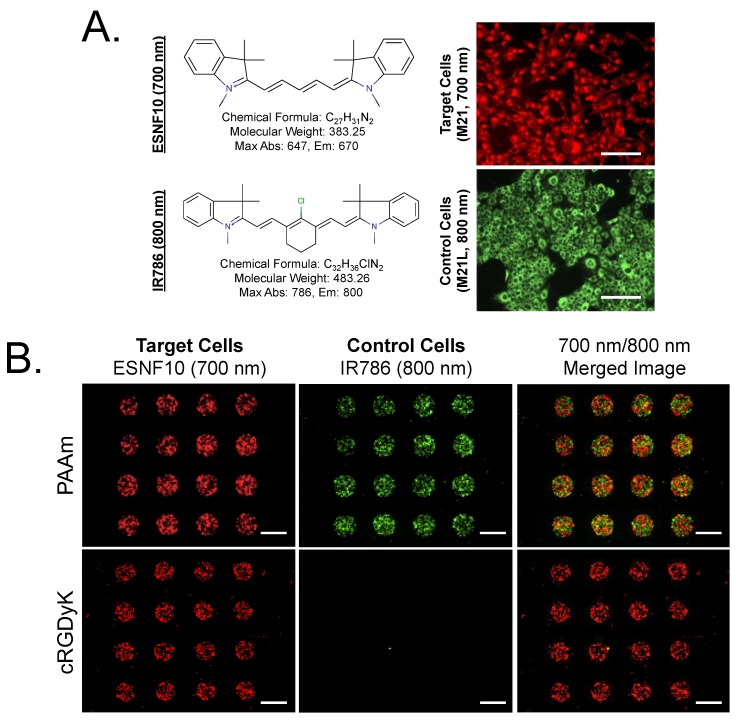
Dual-channel screening strategy and controls. (**A**) Living integrin α_v_β_3_-positive M21 cells (target cells; stained with ESNF10 and pseudo-colored in red) and integrin α_v_β_3_‑negative M21-L cells (control cells; stained with IR786 and pseudo-colored in green) prior to dissociation from their respective plates. Scale bars = 100 μm. (**B**) The same cells mixed together and panned over PAAm positive control spots (top row) or cRGDyK ligand spots (bottom row). The yellow color indicates co-localized M21 and M21-L cells. Scale bars = 300 μm.

To validate the assay, integrin α_v_β_3_-positive M21 cells (positive control) labeled with the 700 nm NIR fluorophore ESNF10 and integrin α_v_β_3_-negative M21-L cells (negative control) labeled with the 800 nm NIR fluorophore IR786 were panned over the surface of our SMM (Figure 1A). PAAm, a “sticky” cationic polymer showing no specificity to cell surfaces was used as a positive ligand control, which bound all cell types. Using dual-channel NIR fluorescence microscopy, the number of individual cells binding each spot could be counted (Figure 1B). Thus the readout of our assay was number of cells bound per spot, with the theoretical maximum number of bound cells (*i.e.*, the dynamic range of the assay) being defined by the PAAm control spots (≈300 cells per spot for all cell lines tested).

Results of the assay using the integrin-binding peptide cRGDyK as the ligand spot are shown in Figure 1B. Specificity was defined in one of two ways. In the absence of negative control cells, specificity was the number of receptor-positive cells binding a ligand spot divided by the number of these same cells binding inter-spot blank space on the slide. In the presence of negative control cells, specificity was the number of receptor-positive cells binding a ligand spot minus the number of receptor-negative cells bound to that same spot. Sensitivity was defined as the absolute number of receptor-positive cells bound to a particular spot. Of note, pseudo-coloring of 700 nm fluorescence in red and the 800 nm fluorescence in green permitted rapid visual assessment of specificity as demonstrated in Figure 1.

### 3.2. Optimization of SMM Screening Using Living Cells

**Figure 2:**
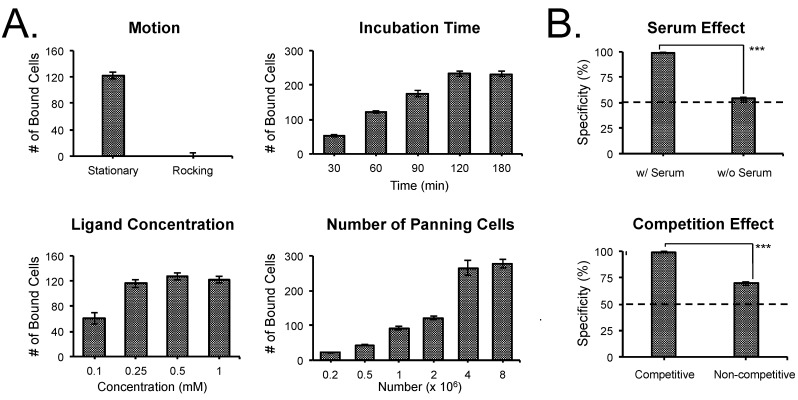
Optimization of screening parameters: (**A**) Maximizing sensitivity through the effect of motion, incubation time, ligand spotting concentration, and the number of panned cells using a cRGDyK array and a mixture of M21 and M21-L cells. Shown are mean ± SD for each data point from 4 randomly chosen spots on the slide. (**B**) Maximizing specificity through the use of serum or competing receptor-negative cells.

In order to optimize screening parameters of our SMM using living cells, cRGDyK spots were arrayed, and a mixture of M21 and M21-L cells were applied while systematically varying motion, incubation time, ligand spotting concentration, and number of panned cells (Figure 2A). Notably, cell binding was greatly improved in the absence of motion, a result that might be explained by a boundary layer of shear stress created immediately above the surface of the slide in the presence of motion. Once stationary, cell binding increased linearly with incubation time up to 120 min, at which time saturation occurred. Cell binding also increased as a function ligand spotting concentration, with saturation occurring above 0.25 mM. The number of panned cells also increased binding, with saturation occurring at 4 × 10^6^ cells per slide. To maximize the specificity of binding, we compared incubation in the presence or absence of 10% serum, and with or without negative control cells (Figure 2B). Both serum and competing cells improved specificity, likely by blocking non-specific interactions.

### 3.3. Screening of Diverse Chemical and Cellular Interactions

**Figure 3:**
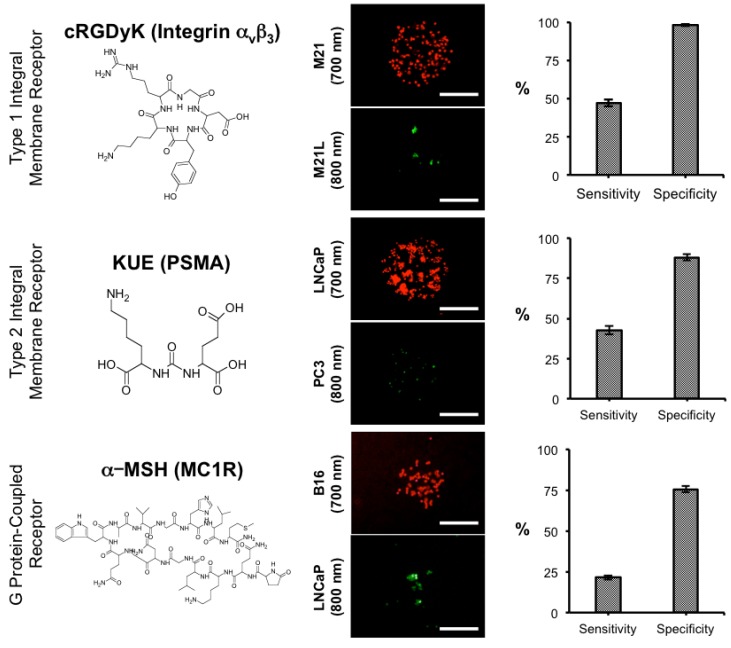
Robustness of the SMM Screening Assay: Three different model systems, varying in ligand chemical structure, cell type, receptor transmembrane topology, B_max_, and ligand affinity were tested as described in the text. Shown are mean ± SD for each data point from four randomly chosen spots on the slide. Receptor-positive and receptor-negative cells were labeled with 700 nm and 800 nm NIR fluorophores and pseudo-colored red and green, respectively, during microscopy. Scale bars = 200 μm.

To explore the usefulness of our SMM screening system, we tested three cell-ligand interactions, which all differed in terms of cell type, receptor type, ligand type, and B_max_ (*i.e.*, the number of receptors per cell) and are shown in Figure 3 and summarized in Table 1. M21 cells have approximately 5 × 10^4^ integrin α_v_β_3_ receptors per cell (*i.e.*, B_max_) on their surface, while M21-L cells have no detectable integrin α_v_β_3_ receptors (1 × 10^3^) [27]. Integrin α_v_β_3_ has a type I transmembrane topology and binds the cyclic peptide ligand cRGDyK with an affinity of approximately 50 nM. LNCaP cells have approximately 2 × 10^5^ prostate-specific membrane antigen (PSMA) receptors per cell on their surface, while PC3 cells have no detectable PSMA [28]. PSMA has a type II transmembrane topology and binds the small molecule KUE with an affinity of approximately 15 nM. B16 cells have approximately 7 × 10^3^ melanocortin 1 receptors (MC1R) receptors per cell on their surface, while LNCaP cells have no detectable MC1R [29]. MC1R is a G protein-coupled receptor with 7 transmembrane domains and binds the peptide α-melanocyte stimulating hormone (α-MSH) with an affinity of approximately 0.4 nM. Using the optimized parameters from Figure 2 (no motion, 60 min incubation time, 1 mM ligand spotting concentration, and 4 × 10^6^ panned cells per slide), all three cell-ligand interactions are detectable with our SMM screening system with relatively high specificity and with sensitivity proportional to B_max_.

It should be noted, however, that many of the system parameters are inter-dependent and should be re-optimized for a particular model system. For example, in the presence of extremely high affinity and B_max_, motion might not only be possible but could improve specificity by reducing non-specific interactions. Similarly, we only explored the presence or absence of 10% serum, but in some model systems, a higher or lower concentration could be optimal.

**Table 1:** Selected small molecule ligands and cell lines used for SMM screening.

Compound	M.W.(Da)	Affinity (*K*_D_)	Specificity (Receptor)	Tested Cells (Positive/Negative)	Ref.
β-AG	248.19	2 µM	PSMA	LNCaP/PC3	[22,23]
β-AG trimer	1213.20	60 nM	PSMA	LNCaP/PC3	[22,23]
GPI	311.23	9 nM	PSMA	LNCaP/PC3	[22,23]
GPI trimer	1360.23	0.7 nM	PSMA	LNCaP/PC3	[22,23]
KUE	319.31	15 nM	PSMA	LNCaP/PC3	[28]
cRGDyK	619.67	50 nM	Integrin α_v_β_3_	M21/M21-L	[30]
α-MSH	1664.88	0.4 nM	MC1R	B16/LNCaP	[29]
PAAm	~70,000	N.A.	Nonspecific	All Cells	[21]

β-AG, beta Ala-Gly; cRGDyk, cyclo Arg-Gly-Asp-D-Tyr-Lys; GPI, 2[(3-amino-3-carboxypropyl)(hydroxy)(phosphinyl)-methyl]pentane-1,5-dioic acid; KUE, 2-(3-(5-amino-1-carboxypentyl)ureido)pentanedioic acid; MC1R, melanocortin 1 receptors; α-MSH, α-melanocyte stimulating hormone; M.W., molecular weight; N.A., not applicable; PAAm, polyallrylamine; PSMA, prostate-specific membrane antigen.

### 3.4. Exploring the Relationship between Ligand Affinity and B_max_

**Figure 4:**
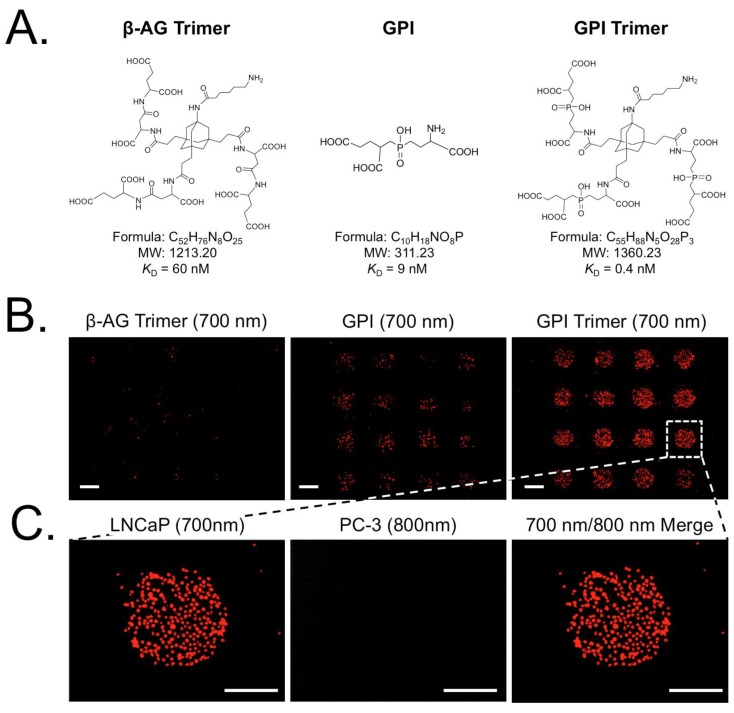
The effect of affinity on cell-ligand spot binding: PSMA-positive LNCaP cells and PSMA-negative PC3 cells were labeled with 700 nm and 800 nm NIR fluorophores and pseudo-colored in red and green, respectively. (**A**) Chemical structures of targeting ligands employed, (**B**) 4× microscopy images, and (**C**) 20× microscopy images. Scale bars = 200 μm.

The results from Figure 3 suggested that ligand affinity (*K*_D_), and therefore the ratio of affinity to B_max_, might have a profound impact on the sensitivity of the SMM screening assay. To explore this relationship we utilized a series of PSMA ligands previously reported by our group (Figure 4A), which spanned a wide range of affinity [22,31]: β-AG (*K*_D_ = 2 µM), GPI (*K*_D_ = 9 nM), β-AG trimer (*K*_D_ = 60 nM), and GPI trimer (*K*_D_ = 0.4 nM). The SMM was probed with PSMA-positive cells (LNCaP) and PSMA-negative cells (PC3), using the optimized parameters described for Figure 3. As shown in Figure 4, the SMM assay was able to perform well over 3 logs of affinity space, with the number of cells bound per spot being proportional to affinity. These results reinforce the importance of defining any cell-bound ligand spot as “positive” during initial screening of diverse chemical libraries because low affinity ligands might have only a few cells bound. And, if B_max_ is low, even high affinity interactions may result in only a few cells bound per spot [22,31].

## 4. Conclusions

We have developed a SMM glass slide-based system for the rapid and efficient screening of ligands that bind to the surface of living mammalian cells. If careful attention is paid to critical parameters, the system appears to function well over a wide range of ligand affinities, receptor topologies, and cell types of interest. It is hoped that this system can be applied to the screening of diverse and complex chemical libraries to find lead candidates for improved diagnostic and therapeutic drugs.
